# Pure Thalamic Infarct: 8-Year Follow-Up Study in a Hospital in China

**DOI:** 10.3389/fneur.2021.715317

**Published:** 2021-09-14

**Authors:** He Liang, Anand Karthik Sarma, Zhenxing Wang, Ming Mo, Jianwen Lin, Xunming Ji, Dong Chen, Yi Liu

**Affiliations:** ^1^Department of Neurology, Dalian Municipal Central Hospital, Affiliated Hospital of Dalian Medical University, Dalian, China; ^2^Department of Neurology, Wake Forest University, Winston-Salem, NC, United States; ^3^Department of Neurosurgery, Xuanwu Hospital, Capital Medical University, Beijing, China

**Keywords:** pure thalamic infarct, etiology, blood supply, 8-year follow-up, comorbidity

## Abstract

Pure thalamic infarct is a rare lacunar stroke type, with little known about long-term outcomes. This 8-year, single-center, retrospective study evaluated the clinical background, etiology, Trial of ORG 10172 in Acute Stroke Treatment (TOAST) classification, and 8-year follow-up results in 27 patients with pure thalamic infarcts identified by MR diffusion-weighted imaging in Dalian, China. All patients presented chief complaints of limb weakness or sensory disturbances. Hypertension (24/27, 88.9%), diabetes (12/27, 44.4%), atrial fibrillation (1/27, 3.7%), hyperlipidemia (10/27, 37%), hyperhomocysteinemia (6/27, 22.2%), smoking history (10/27, 37%; 9/15, 60% for men; 1/12, 8.3% for women), and excessive alcohol consumption history (7/27, 25.9%; 7/15, 46.7% for men; 0 for women) were observed in our patient population. Based on TOAST classification, 1 patient had large artery atherosclerosis (7.14%), 23 had small vessel occlusion (SVO; 85.2%), and 3 patients were unidentified due to lack of cerebral angiography. The thalamic blood supply classification were as follows: 23 (85.2%), inferolateral territory; 1 (3.7%), tuberothalamic territory; 2 (7.4%), combination of tuberothalamic and paramedian arteries; 1 (3.7%), combination of inferolateral and paramedian arteries; 0, posterior choroidal arteries. During the 8-year follow-up, 3 patients died of colon cancer, multi-organ failure, and kidney failure, respectively; 7 presented with a recurrent stroke; while 10 recovered well with their risk factors under control. In conclusion, our cohort of pure thalamic infarcts were mainly due to SVO (TOAST), with hypertension as the main risk factor, and the inferolateral artery as the most implicated arterial territory. Less severe outcome or stroke recurrence are identified in long-term follow-up of pure thalamic infarcts. Other comorbidities would be cause of death in aged patients.

## Introduction

The thalamus contains multiple cerebral nuclei and axonal tracts, which make clinical manifestations more complicated with a variant blood supply. Studies have reported on thalamic blood supplies and their corresponding clinical manifestations of stroke (1, 2). Pure thalamic infarct is a rare lacunar stroke type. And except for case reports, studies on etiology or long-term outcomes have been rarely reported (3). Therefore, this single-center, retrospective study evaluated pure thalamic infarct in the Dalian region in China and analyzed the clinical background, etiology of Trial of Org 10172 in acute stroke treatment (TOAST) classification, and 8-year follow-up results.

## Materials and Methods

### Patients

A total of 1,179 patients with abnormal diffusion-weighted imaging (DWI), which was finished 7 days after admission to the Inpatient Department of Dalian Municipal Central Hospital, were screened between January 2013 and December 2014. Pure thalamic infarct was confirmed by DWI with an ischemic region restricted to four groups of the thalamic blood supply territory (1, 4, 5). Exclusion criteria were partial or total top of the basilar syndrome and a combination of thalamic and other anterior circulation territories. Other etiologies of metabolism, infection, inflammation, or neoplasm to cause thalamic lesions in DWI were also excluded during inpatient and follow-up period.

### Clinical Information, Imaging Analysis, and Additional Examinations

Clinical information was recorded as follows: chief complaints; age; sex; a history of hypertension, diabetes, smoking, and excessive drinking; and National Institute of Health Stroke Scale score.

Each patient underwent the following examinations: blood hepatic function, kidney function, creatine kinase, creatine kinase-MB, cardiac troponin T (cTnT), lipid (total cholesterol, triglyceride, or low-density lipoprotein), homocysteine, coagulation function [prothrombin time (PT), international normalized ratio (INR), activated partial thromboplastin time (APTT), fibrinogen (FIB), thrombin time (TT), and D-Dimer (DD)], C-reactive protein (CRP), erythrocyte sedimentation rate (ESR), ECG, ultrasound (heart, carotid artery, low extremities), and cerebral magnetic resonance angiography (MRA) or computed tomography angiography (CTA). If cardioembolism was highly suspected, 24-h Holter, transesophageal echocardiogram, and agitated saline contrast echocardiography would be performed. For rare cases, immunologic tests would be further used for etiology classification. To achieve blind design, different groups of researchers performed clinical background evaluation, radiologic imaging analyses, etiology classification, and outcome follow-up separately.

**Figure 1:**
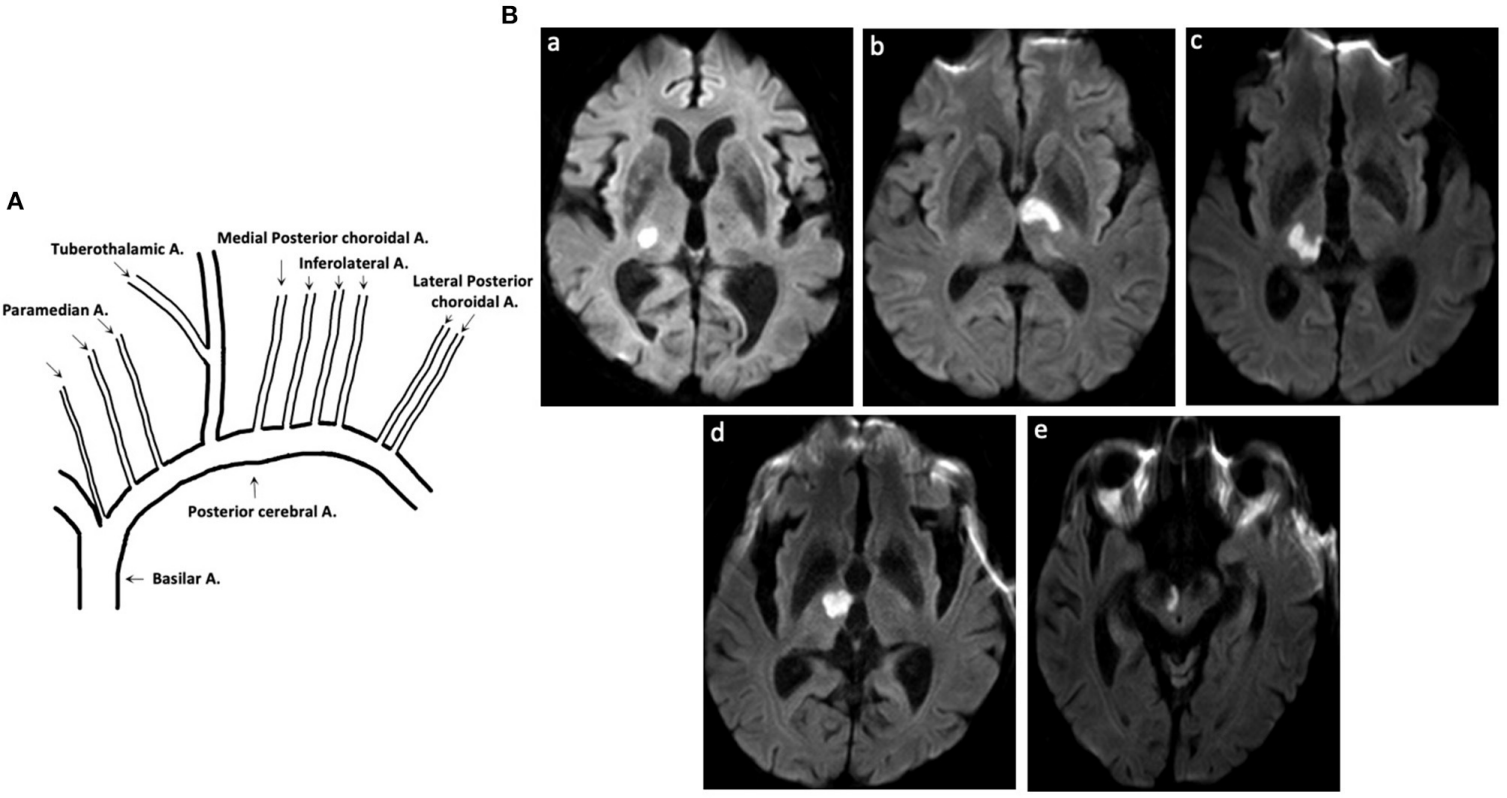
Thalamic blood supplies and DWI of patients with acute pure thalamic infarct. **(A)** Thalamic blood supplies. **(B)** DWI-proven acute pure thalamic infarct is shown in the territory of the (a) inferolateral artery, (b) tuberothalamic artery, (c) combination of inferolateral and paramedian arteries, and (d,e) combination of tuberothalamic and paramedian arteries. A, artery; DWI, diffusion-weighted imaging.

Thalamic blood supplies were classified into four groups: inferolateral, tuberothalamic, paramedian, and posterior choroidal arteries (1). TOAST etiology were classified following the above mentioned prognostic procedure as follows: large artery atherosclerosis (LAA), cardioembolism (CE), small vessel occlusion (SVO), other determined etiology, and undetermined etiology (6) (Figure 1).

In the 8-year follow-up questionnaire, the causes of death and stroke recurrence were recorded.

### Statistical Analysis

Clinical background evaluation, radiologic imaging analyses, and all other examinations were performed by trained neurologists blinded to the patient's identity, and statistical analysis was performed using SPSS version 17.0 (SPSS Inc., Chicago, IL, USA). Age and the average onset-to-door (OTD) time between men and women were subjected to Student's *t*-test. Differences were considered statistically significant at *p* < 0.05. A biostatistician was consulted to ensure appropriate statistical testing throughout the study.

## Results

### Clinical Background

In total, 12 women and 15 men were enrolled (mean age: 68 vs. 62.9 years, *p* = 0.24) with chief complaints of limb weakness or sensory impairment. The clinical characteristics are shown in Table 1. The incidence of risk factors was as follows: hypertension (23/27, 85.2%), diabetes (13/27, 48.1%), atrial fibrillation (1/27, 3.7%), hyperlipidemia (10/27, 37%), hyperhomocysteinemia (6/27, 22.2%), a history of smoking (9/27, 33.3%; 9/15, 60% for men and 1/12, 8.3% for women), and a history of excessive drinking (7/27, 25.9%; 7/15, 46.7% for men and 0/12, 0% for women). Hyperlipidemia included increased serum cholesterol, triglyceride, or low-density lipoprotein levels. The average OTD time was 23 h and 51.6 h for women and men, respectively (*p* = 0.190).

**Table 1:** Clinical background, TOAST subtype, and 8-year follow-up results of patients with acute pure thalamic infarcts.

	**Gender**	**Age**	**Smoking**	**Excessive drinking**	**HBP**	**DM**	**HCY**	**HL**	**AF**	**NIHSS**	**TOAST**	**Involved thalamic blood supply**	**8-year follow-up**
1	M	81	+	–	–	+	–	–	–	3	SVO	I	Recurrent stroke
2	M	52	+	+	+	–	+	+	–	1	SVO	I	Well
3	M	46	–	+	+	+	–	+	–	1	SVO	I	Recurrent stroke
4	M	66	–	–	+	–	+	–	–	1	SVO	I	Recurrent stroke
5	M	60	+	**–**	+	–	**–**	–	–	3	SVO	T	Recurrent stroke
6	M	61	+	+	+	–	–	–	–	1	SVO	I	-
7	M	54	–	–	+	+	–	–	–	1	SVO	I	Well
8	M	55	+	+	+	+	–	+	–	1	SVO	I	Well
9	M	84	–	+	+	–	–	–	–	2	SVO	I	Well
10	M	50	–	–	+	–	–	–	–	0	SVO	I	-
11	M	54	+	–	+	–	+	–	–	2	SVO	I	-
12	M	59	+	+	+	+	+	+	–	5	SVO	I	Recurrent stroke
13	M	83	+	–	+	–	+	–	–	2	LAA	T+P	Died, colon cancer
14	M	61	+	+	–	+	–	–	–	3	–	I	-
15	M	68	–	–	+	+	–	+	–	1	SVO	I+P	Died, chronic kidney failure
16	F	88	–	–	+	–	–	–	+	5	–	I	Died, undetermined reason
17	F	50	–	–	+	–	–	–	–	1	SVO	I	Well
18	F	77	–	–	+	+	–	–	–	3	SVO	I	Recurrent stroke
19	F	60	–	–	+	–	–	+	–	3	SVO	I	Well
20	F	78	–	–	+	+	–	–	–	5	SVO	I	Well
21	F	48	–	–	+	–	–	–	–	3	SVO	I	-
22	F	74	–	–	+	+	–	+	–	6	SVO	T+P	Well
23	F	69	–	–	+	+	+	+	–	2	SVO	I	-
24	F	61	–	–	+	–	–	+	–	1	SVO	I	Well
25	F	70	–	–	–	–	–	–	–	5	–	I	Well
26	F	64	–	–	+	+	–	–	–	2	SVO	I	Recurrent stroke
27	F	75	+	–	–	+	–	+	–	5	SVO	I	-

*HTN, hypertension; DM, diabetes mellitus; HCY, hyperhomocysteinemia; HL, hyperlipidemia; AF, atrial fibrillation; NIHSS, National Institute of Health Stroke Scale; TOAST, Trial of Org 10172 in acute stroke treatment; SVO, small vessel occlusion; LAA, large artery atherosclerosis; I, inferolateral artery; T, tuberothalamic artery; P, paramedian artery*.

Thalamic blood supplies were analyzed according to the infarct territory based on previous reports (5). The incidence of involved arteries was as follows: 23 (85.2%), inferolateral territory; 1 (3.7%), tuberothalamic territory; 2 (7.4%), combination of tuberothalamic and paramedian arteries; 1 (3.7%), combination of inferolateral and paramedian arteries; 0, posterior choroidal arteries.

### Stroke Etiology and Follow-Up Results

In this study, 24 of the 27 patients were confirmed by cerebral CTA or MRA. According to the TOAST, stroke etiologies were confirmed as follows: 23, SVO and 1, LAA.

For the 8-year follow-up, 20 of 27 patients were successfully contacted to administer a telephonic questionnaire. Three patients died of rectal cancer (age: 83 years), kidney failure (age: 68 years), and multi-organ failure with unknown cause (age: 81 years). Seven had recurrent stroke. Ten patients recovered well and had mild sensory impairment. Among the four patients aged >80 years, three had tumors: rectal cancer, lymphoma, and pulmonary adenocarcinoma (data not shown).

## Discussion

Thalamic vascular supplies and clinical manifestations of stroke have been well-described in reports. However, little information is available on the long-term outcomes. This study was designed to assess the main risk factors, stroke etiologies, and long-term outcomes of Chinese patients with pure thalamic infarct. The inferolateral artery was the most vulnerable in all thalamic infarcts in this study. Women had a shorter OTD time than men, indicating that men paid less attention to their health in China, and most patients with thalamic infarcts recovered well and only mild sensory impairment remained. Death occurred in older patients owing to complications.

Hypertension, the most important risk factor and resulting in hyaline or hyperplastic arteriolosclerosis, is the main cause of stroke as SVO in the TOAST (7). Lacunar stroke accounts for 15–25% of all first-ever infarcts (8, 9). Pure thalamic infarct is reported to account for 3–4% of cerebral infarcts (10). Information on anatomy, clinical manifestations, and radiology have been reported (1, 4, 11). However, the incidence of thalamic infarct among all strokes or lacunar strokes is rarely reported. In this study, 27 of the 1,179 patients with DWI-proven infarct were confirmed to have thalamic infarcts. The incidence of thalamic infarct among all infarcts and lacunar strokes was 2.3 and ~9–15%, respectively. The ratio of the four groups of thalamic blood supplies in thalamic stroke is controversial. We identified the inferolateral artery as the most vulnerable in all thalamic infarcts, followed by the tuberothalamic artery. An ischemic infarct within the singular paramedian artery or posterior choroidal artery (PCoA) was not identified. A study based on European and American populations revealed that 30–40% of the posterior communicating artery did not contribute to the thalamic supply, and in these instances, the polar territory was alternatively supplied by the paramedian thalamic arteries (12); we confirmed a similar situation in 2 of the 20 thalamic infarcts in our study. Interestingly, singular paramedian artery infarcts were rarely identified in this study, possibly owing to race-induced anatomical differentiation; this aspect should be confirmed in a larger population in the future.

Furthermore, 23 of the 24 thalamic infarcts were confirmed as SVO in the TOAST, and 1 was confirmed as LAA. The current cerebral MRA and CTA technologies could clearly identify atherosclerosis at the origin of the penetrating artery. With increased use of 7T MRI in the future, more SVO will probably be classified as LAA. Thalamic blood supplies originated from the PCA or PCoA perpendicularly, which reduced the possibility of cardiac emboli deposition. Previous studies have reported that 22–35% of all thalamic infarcts were within paramedian territories, with cardioembolism as the main reason (13). The singular paramedian artery was rarely identified in the present study. The TOAST subtype of CE is also rare. Furthermore, other etiologies should be considered if patients with acute infarct do not have the above mentioned risk factors and present with the following characteristics: hereditary stroke background, migraine, recurrent stroke, susceptibility-weighted imaging–proved multiple microbleeds, or temporal lobe white matter hyperintensity. Accordingly, the etiologies of cerebral amyloid angiopathy; hereditary small vessel disease (cerebral autosomal dominant arteriopathy with subcortical infarcts and leukoencephalopathy); cerebral autosomal recessive arteriopathy with subcortical infarcts and leukoencephalopathy; Fabry disease; mitochondrial encephalomyopathy, lactic acidosis, and stroke-like episodes; venous collagenosis; immune-inflammation-mediated vasculitis; and radiation exposure should be considered (7, 14).

The anterior choroidal artery (AChA) infarct is most easily misrecognized as a thalamic blood supply infarct. AChA, originating from the terminal internal cerebral artery, affects the posterior limb of the internal capsule, lateral geniculate, thalamus, uncus, or cerebral peduncle (15). In collaboration with the PCoA, the AChA, and PCoA territories closely overlap (16).

Patients enrolled in this study visited our hospital because of limb weakness or sensory impairment, which indicated that less attention was paid to other atypical symptoms of thalamic infarcts, including decreased consciousness, learning and memory impairment, neuropsychological disturbances (disorientation, unconcern, apathy, and amnesia), visual dysfunction (total or partial oculomotor nerve palsy, vertical gaze, and visual defect), language dysfunction (aphasia and dysarthria), dystonia, and tremors.

There are two main limitations of this study. First, this was a single-center study that enrolled patients with pure thalamic infarct without the involvement of the top of the basilar artery or other anterior circulation territories, which limited the number enrolled, missed those patients with rare variations in thalamic blood supplies. Thus, multi-center studies would be highly recommended. Second, angiography with higher resolution, such as digital subtraction angiography or 7T MRI, was not used. The TOAST subtype of LAA was misclassified as SVO. Proximal part of bifurcation is most susceptible to atherosclerosis. Inferolateral artery originates nearest to bifurcation of two PCAs, which might explain vulnerability of inferolateral artery in pure thalamic infarct. Therefore, we suggest an oral intake of statins and maintaining normal liver function even in patients without hyperlipidemia. More studies on thalamic infarct in a larger Chinese population are needed in the future.

Many patients with thalamic infarcts in this study present anxiety or even fear about unknown future recovery or recurrence. However, thalamic infarcts did not cause a disastrous outcome in long-term follow-up. Comorbidities in aged patients, such as tumors and failure of main organs, acted as main cause of death. Besides, prevention therapy based on stroke etiology should be well-recognized and controlled.

In conclusion, our cohort of pure thalamic infarcts were mainly due to SVO (TOAST), with hypertension as the main risk factor, and the inferolateral artery as the most implicated arterial territory. Less severe outcome or stroke recurrence are identified in long-term follow-up of pure thalamic infarcts. Other comorbidities would be cause of death in aged patients.

## Data Availability Statement

The original contributions presented in the study are included in the article/supplementary material, further inquiries can be directed to the corresponding authors.

## Ethics Statement

The studies involving human participants were reviewed and approved by Dalian Municipal Central Hospital. The patients/participants provided their written informed consent to participate in this study.

## Author Contributions

All authors listed have made a substantial, direct and intellectual contribution to the work, and approved it for publication.

## Funding

This work was supported by the LiaoNing Revitalization Talents Program (grant number XLYC 1807083), National Natural Science Foundation of China (Grant No. 81200915), and Dalian Science Innovation Project (Grant No. 2021JJ13SN64).

## Conflict of Interest

The authors declare that the research was conducted in the absence of any commercial or financial relationships that could be construed as a potential conflict of interest.

## Publisher's Note

All claims expressed in this article are solely those of the authors and do not necessarily represent those of their affiliated organizations, or those of the publisher, the editors and the reviewers. Any product that may be evaluated in this article, or claim that may be made by its manufacturer, is not guaranteed or endorsed by the publisher.

## Abbreviations

AChA: anterior choroidal artery
TOAST: Trial of Org 10172 in acute stroke treatment
CS: cardiogenic stroke
CTA: computed tomography angiography
DWI: diffusion-weighted imaging
LAA: large artery atherosclerosis
MRA: magnetic resonance angiography
OTD: onset-to-door
SVO: small vessel occlusion
PCoA: posterior choroidal artery.
